# A Digital-Analog Hybrid System-on-Chip for Capacitive Sensor Measurement and Control

**DOI:** 10.3390/s21020431

**Published:** 2021-01-09

**Authors:** Zhenyi Gao, Bin Zhou, Xiang Li, Lei Yang, Qi Wei, Rong Zhang

**Affiliations:** 1 Engineering Research Center for Navigation Technology, Department of Precision Instrument, Tsinghua University, Beijing 100084, China; gaozy17@mails.tsinghua.edu.cn (Z.G.); li-x07@mails.tsinghua.edu.cn (X.L.); rongzh@mail.tsinghua.edu.cn (R.Z.); 2 College of Information Science and Engineering, Shandong Agricultural University, Tai’an 271018, China; yanglei@sdau.edu.cn

**Keywords:** capacitive sensors, signal processing, SoC, low power, miniaturization

## Abstract

Sensors based on capacitance detection are common in the field of inertial measurement and have the potential for miniaturization and low power consumption. In order to control and process such sensors, a novel digital-analog hybrid system-on-chip (SoC) is designed and implemented. The system includes a capacitor to voltage (C/V) conversion circuit and a band-pass sigma-delta modulator (BPSDM) as the analog-to-digital converter (ADC). The digital signal is processed by the dedicated circuit module based on the least mean square error demodulation (LMSD) algorithm on the chip. The low-power Cortex-M3 processor supports software implementation of control algorithms and circuit parameter configuration. The control signal is output through a digital BPSDM. The chip was taped out under SMIC 180 nm Complementary Metal Oxide Semiconductor (CMOS) technology and tested for performance. The result shows that the maximum operating frequency of the chip is 105 MHz. The total area is 77.43 mm^2^. When the system clock is set to 51.2 MHz, the static power consumption and dynamic power consumption of the digital system are 18 mW and 54 mW respectively.

## 1. Introduction

The measurement of physical quantities based on capacitance detection has the characteristics of low cost, miniaturization, and high accuracy. This detection scheme is commonly used in the field of inertial measurement such as gyroscopes, accelerometers [1,2], and angular displacement sensors [3]. The use of this type of sensor needs to be provided with an analog control signal. After the value of the capacitance is detected, it is converted into a digital quantity through the C/V circuit and the ADC circuit and is demodulated according to the signal modulation method [4]. The processing circuit discussed was firstly built using discrete devices. In order to achieve higher system integration, low power consumption, and to has the ability to apply complex processing algorithms, the digitalization of processing circuits is getting higher, and application-specific integrated circuits (ASIC) have become a development trend to replace discrete devices [5].

The ASIC implementation of the analog part of the processing circuit is relatively mature at present. Hou et al. reported in 2019 an analog interface ASIC for a capacitive angle encoder, which consists of a C/V converter and an ADC [6]. In 2019, Lv et al. also presented an analog interface ASIC which consists of a C/V converter and a BPSDM ADC [7], and the circuit is used for micro-electromechanical systems (MEMS) vibratory gyroscopes. In 2020, Ju et al. reported an auto-tuning continuous-time BPSDM for capacitance signal detection of MEMS gyroscopes [8]. Similar interface circuits [2,9,10] differ mainly in C/V converter and ADC. In the field of inertial measurement, interface ASIC based on capacitance detection continue to improve performance in terms of noise floor, dynamic range, and signal-to-noise ratio.

The digital signal processing involved in the measurement and control circuit was originally implemented in discrete devices. The digital phase demodulation circuit of the gyro signal implemented on the Printed Circuit Board (PCB) mentioned in [11] is a typical example. Using a digital signal processor (DSP) to realize the control algorithm in software, the demodulation method has higher flexibility and reliability. The MEMS inertial measurement unit (IMU) introduced by Geiger et al. [12] is based on the abovementioned scheme for signal processing. Signal processing on a DSP has issues about phase mismatch and signal real-time performance. Implementing signal processing algorithms on a Field Programmable Gate Array (FPGA) at the circuit level can solve the above problems and become a mainstream solution. In related works [7,10,13,14,15,16], the researchers reported their cases of signal measurement and control based on FPGA in the field of inertial measurement. When the digital signal processing circuits are designed and manufactured using integrated circuit technology, it can obtain performance beyond the FPGA solutions, which are also reported in some products [12,17].

In order to cope with different usage scenarios and combine control and compensation algorithms, a development trend of digital integration solutions is to use SoC solutions to implement software-defined circuit parameters and structures. In addition, the digital-analog hybrid system-on-chip can bring higher integration and lower the cost [5]. In practical applications, sensor information fusion is very extensive, and the design of a single measurement and control chip compatible with multiple sensors is also a development trend, which plays an important role in the development of miniaturization and low power consumption of terminal equipment. Based on mainstream solution, which is consisting of an analog ASIC interface circuit and an FPGA to implement digital algorithms, the proposed solution in this paper is to integrate the two parts in a single chip, which further improves the system integration. In terms of compatibility, this paper proposed a general signal model and described the circuit-level architecture design, which realizes one chip to deal with different sensor signals. The proposed design will enable the chip has the ability to switch the working state or to work in different states at the same time.

Taking capacitive sensors in the field of inertial measurement as the research objects, this paper reports a digital-analog hybrid SoC with sensor compatibility. The system includes analog C/V converter, BPSDM, digital system based on Cortex-M3 [18] processor, dedicated demodulation circuit based on LMSD algorithm, digital BPSDM and current steering digital to analog converter (DAC). The system includes two signal demodulation channels and two AC control signal output channels. The dedicated demodulation circuit can demodulate digital quantities in the form of trigonometric functions to obtain sensor information. The chip was taped out under SMIC 180 nm technology and tested for performance, and its sensor compatibility was verified in the frequency sweep experiment of a MEMS gyroscope and the demodulation test of an angular displacement sensor. The next section will discuss the description for the architecture of the SoC. Section 3 and Section 4 respectively present the implementation contents of analog integrated circuits and digital integrated circuits. Section 5 provides the experimental results, and conclusions and future research plans are discussed in Section 6.

## 2. Overall Description for the Architecture and Function of the Designed SoC

### 2.1. Description of the Architecture and the Signal Model

**Figure 1:**
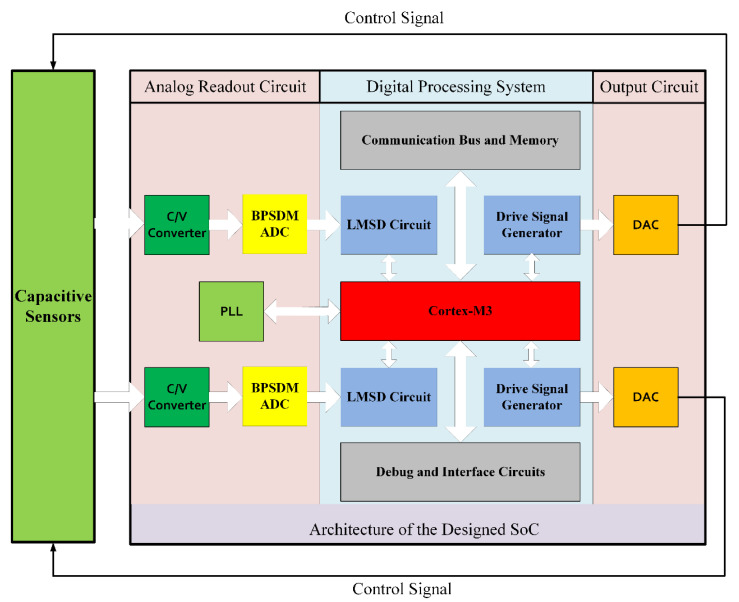
Architecture diagram of the SoC.

The simplified architecture of the designed SoC is shown in Figure 1. The capacitance signal from the sensor is converted into a digital signal by an analog readout circuit, and the measurement information is calculated in the digital processing system. The program running in the processor can configure the parameters of the digital demodulation circuit and the driving circuit, and then output the driving signal in the form of trigonometric function through the DAC.

The system provides two channels for sensor drive and signal detection. The circuit parameters and drive signals can be adjusted by software programs to realize closed-loop control. For the control and measurement of MEMS gyroscopes, accelerometers, and angular displacement sensors, the configuration of the system is sufficient. More signal processing channels will enable a single chip to control more sensors.

The sensors discussed convert the physical quantity that needs to be detected into a change in capacitance, and the change in capacitance has the following form [3,8,10,11,12,13,14,15,16]:

(1) $$\Delta C=A\cdot \mathrm{carr}\left(\omega_{c},\;\varphi_{c}\right)\cdot \sin\left(\omega_{s}\cdot t+\varphi_{s}\right).$$

In the above formula, ΔC represents the change value of the capacitance, carr(∙) is the expression of the carrier in the form of a square wave and $\omega_{c}$ is the frequency of the carrier, and $\omega_{s}$ is the frequency related to the sensor characteristics and signal modulation method. For example, in a gyro, the modulated signal is in the form of a sine function and $\omega_{s}$ represents a resonance frequency of drive axis or detection axis, and the capacitance signal has the form of Equation (1). While in an angular displacement sensor, the modulated signal has a constant value, and the carrier is a sine function. In this case, the capacitance signal has the following form:

(2) $$\Delta C=A\cdot \sin\left(\omega_{s}\cdot t+\varphi_{s}\right).$$

$\omega_{s}$ represents the frequency of the carrier. In the above two formulas, $\varphi_{c}$ and $\varphi_{s}$ represent the relevant phase information. In addition, A represents the magnitude of the capacitance. The value change of A has a linear relationship with the physical quantity to be detected, and the information detected by the sensor can be obtained from it.

### 2.2. Overall Description of the Key Circuits

The two key modules of the analog readout circuit are the C/V converter and the ADC. The former converts the capacitance value into a voltage value, and the latter converts the analog voltage value into a digital value. The details will be described in Section 3.

The key circuit of the digital processing system is the LMSD module, which extracts the amplitude and phase information from the digital signal in the form of Formula (1). According to the signal modulation mode and carrier form of different sensors, the parameter configuration of the LMSD calculation channel can be carried out by the program running in the processor. The software-defined hardware parameters enable a single circuit to process multiple types of sensor signals. When the sensor signal has the form of Formula (2), the common clock gating [19] technology in digital circuit design can turn off the drive clock of the redundant module. The details of the algorithm and the circuits will be described in Section 4.

The drive signal generator outputs a drive signal in the form of a trigonometric function, which is modulated by digital BPSDM [20] and output by a 20-bit current steering DAC [21]. The four-stage cascade-of-integrators feed-forward structure was implemented in the design of the digital BPSDM, and the center frequency of pass band can be configured through software. There is no more novel content in the related design and no more specific introduction in the article.

The Phase Locked Loop (PLL) is a digital-analog hybrid module that can convert the output clock of the crystal oscillator to a stable clock signal. The stable clock signal is used to drive the SoC and the clock frequency can be configured dynamically by software. A dedicated bootloader was integrated into the SoC, which supports program loading through on-chip One Time Programmable (OTP) memory, off-chip flash memory or online Joint Test Action Group (JTAG) debugger. Detailed information about unique designs for a variety of application scenarios has been reported in previous work [22].

In terms of communication bus and memory, Advanced Microcontroller Bus Architecture (AMBA) [23] is used as a communication protocol for the transmission of control signals and data between the processor and the circuit modules. The size of on-chip OTP memory is 128 KB, which is sufficient for inertial measurement needs. In addition, 16 General Purpose Input Output (GPIO) interfaces, 2 serial Universal Asynchronous Receiver/Transmitter (UART) modules, one Serial Peripheral Interface (SPI) and the JTAG interface constitute the entire system debugging interface circuits.

## 3. Design Description of the C/V Converter and the ADC

### 3.1. Implementation Details of the C/V Converter

**Figure 2:**
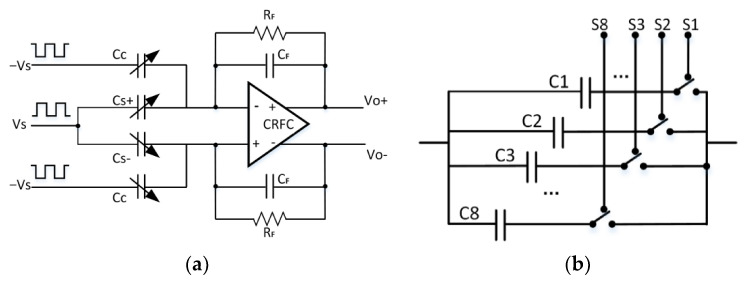
The designed C/V converter based on DC capacitance cancellation. (**a**) Schematic diagram of the charge amplifier for differential capacitance detection; (**b**) DC capacitance cancellation scheme.

A charge amplifier was implemented in the C/V converter. The basic principle of a charge amplifier based on differential capacitance detection is shown in Figure 2a. Using a capacitor as a feedback element, the amplifier’s noise bandwidth can be reduced and the detection accuracy can be improved [24].

As shown in Figure 2a, assume that the capacitance value to be detected is $C_{s}=C_{0}\pm \Delta C$, where $C_{0}$ is the basic capacitance value, ΔC is the change in capacitance value, and $C_{c}$ is the on-chip cancellation capacitance in the circuit. An AC voltage is added at the input end of the differential capacitor as a voltage source, and a reverse voltage source is added at the input end of the cancellation capacitor. According to the amount of charge transfer on the detection capacitor and the cancellation capacitor, the amount of charge change at one input of the charge amplifier is calculated as follows:

(3) $$Q=\Delta \left(C_{s}\times V_{S}\right)+\Delta \left(C_{c}\times (-V_{S})\right)\;=\Delta C_{s}\times V_{s}+C_{s}\times \Delta V_{s}-\Delta C_{c}\times V_{s}-C_{c}\times \Delta V_{s}\;=\pm \Delta C\times V_{s}+\left(C_{0}\pm \Delta C\right)\times \Delta V_{s}-C_{c}\times \Delta V_{s}.$$

When the second-order small quantities are ignored and $C_{c}=\pm C_{0}$, Equation (3) is transformed into the following equation:

(4) $$Q=\pm \Delta C\times V_{s}.$$

The principle of the DC cancellation capacitor $C_{c}$ is shown in Figure 2b) The voltage source is a carrier with the opposite phase to the high-frequency carrier, and the internal structure is a DC capacitor array, including eight cancellation capacitors and switches. The eight CMOS switches S1, S2, S3,..., S8 are controlled by the digital system to determine the value of $C_{c}$ is $2^{n}\cdot C$ (n = 1, 2,…, 8). After the processing of the capacitive inertial sensor is completed, $C_{0}$ has been determined, and this method can correct part of the DC error of the sensor. In this design, the value of the basic capacitance C is 49 fF.

The complementary recycling folded cascode (CRFC) architecture [6] was adopted as the amplifier in the C/V conversion circuit. Combined with Equation (3), the output transfer function of the charge amplifier is as follows:

(5) $$V_{o+}=\frac{-j\omega }{\frac{1}{R_{F}}+j\omega C_{F}}\times \left(\Delta C\times V_{s}\right),\;V_{o-}=\frac{-j\omega }{\frac{1}{R_{F}}+j\omega C_{F}}\times \left(-\Delta C\times V_{s}\right).$$

According to Equation (5), the C/V circuit converts the capacitance change value into a voltage value with a linear change relationship. The existence of the cancellation capacitor can suppress the DC error and improve the gain coefficient of the conversion circuit.

### 3.2. Implementation Scheme of the BPSDM ADC

**Figure 3:**
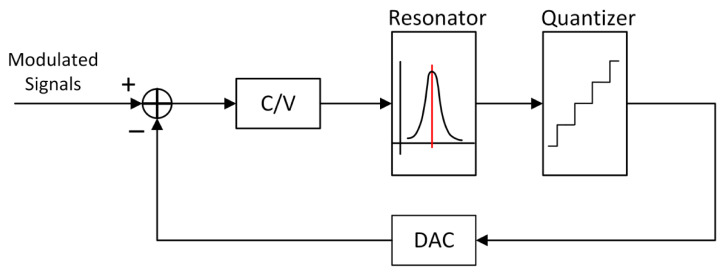
Schematic diagram of the BPSDM circuit.

According to Equation (1), the sensor signal is modulated to high frequency, and a band pass ADC is applied, which can effectively reduce the impact of the low frequency noise of the operational amplifier on the circuit. The schematic diagram of the BPSDM circuit is shown as in Figure 3. The adopted solution is a continuous-time third-order band pass modulator, in which a resonator, a quantizer and a DAC are included. The resonator is mainly composed of RC filter structure [8], which provides noise shaping capability for ADC. The quantizer with successive approximation structure outputs a 3-bit digital code stream, which is used to provide a quantized digital signal and a feedback signals. A capacitive feedback array is implemented in the DAC feedback network. The quantized signals are fed back to the input of the C/V module, and negative feedback charge is injected at the feedback point to offset the charge change caused by the sensor capacitance change, forming a negative feedback analog signal and realizing a switched capacitor DAC

**Figure 4:**
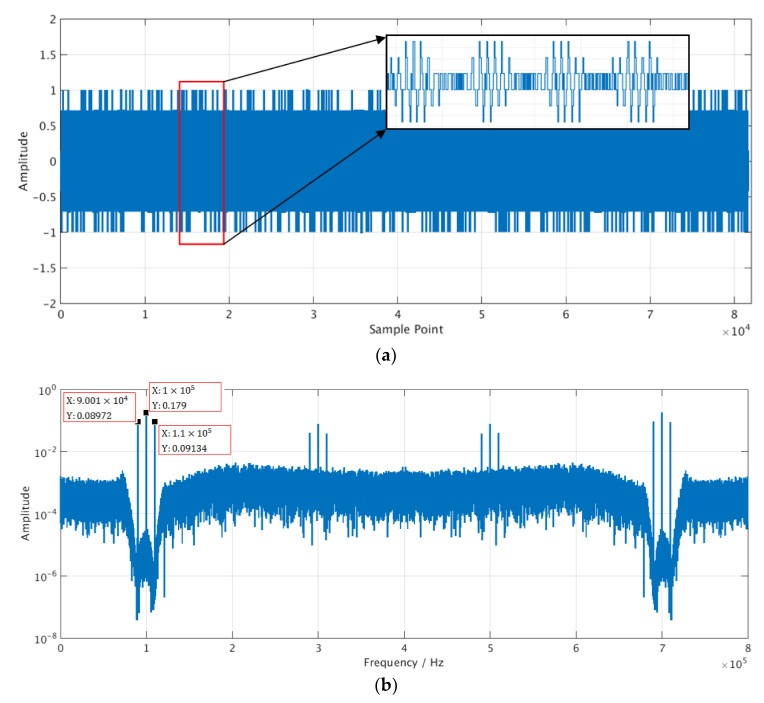
Simulation data of analog readout system. (**a**) Time-domain waveform of analog circuit output signal; (**b**) amplitude spectrum of the signal.

The circuit of the analog readout system was implemented in Cadence, which is a software for integrated circuit design and simulation. The carrier frequency was set to 100 kHz and the signal frequency was set to 10 kHz for simulation. According to Equation (1), the simulated input signal was obtained and the simulated output signal was collected. As show in Figure 4, with signal modulating and noise shaping, the shape of the original signal is not clear in the time domain waveform of the output 3 bit digital signal. In the frequency domain, the noise floor near the signal frequency point is low, and the three spectral lines of the carrier and the signal can be clearly distinguished.

The digital signal output by the analog readout circuit is demodulated in the digital processing system, which is introduced in the next section. The amplitude of each component in the modulated signal and the signal amplitude to be measured will be calculated in the digital circuit and output or further processed in Cortex-M3 processor.

## 4. Design Description of Digital Demodulation Module

The digital signal processing circuit demodulates the signal to obtain the sensor signal. The methods used for signal demodulation mainly include multiplication demodulation [25], LMSD and other optimized demodulation algorithms [26,27]. Among these methods, the calculation speed of LMSD is fast and easy to implement in circuit, and optimized demodulation solution based on LMSD is adopted in the circuit.

**Figure 5:**
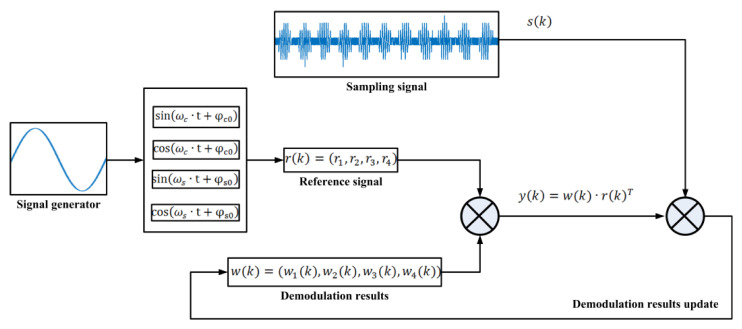
Block diagram of the demodulation method.

The principle of the demodulation scheme is shown in Figure 5. As shown in Figure 4b and Equation (1), the signal processed in the circuit is modulated twice at most. In the time domain, with the multiplication operation of the two frequency signals, the following form of reference signals are generated in the circuit:

(6) $$r_{1}\left(k\right)=\cos\left(\omega_{c}\cdot t+\varphi_{c0}\right)\times \sin\left(\omega_{s}\cdot t+\varphi_{s0}\right),\;r_{2}\left(k\right)=\cos\left(\omega_{c}\cdot t+\varphi_{c0}\right)\times \cos\left(\omega_{s}\cdot t+\varphi_{s0}\right),\;r_{3}\left(k\right)=\sin\left(\omega_{c}\cdot t+\varphi_{c0}\right)\times \sin\left(\omega_{s}\cdot t+\varphi_{s0}\right),\;r_{4}\left(k\right)=\sin\left(\omega_{c}\cdot t+\varphi_{c0}\right)\times \cos\left(\omega_{s}\cdot t+\varphi_{s0}\right).$$

where $\omega_{c}$ and $\omega_{s}$ indicate the frequency related to the modulated signal, $\varphi_{c0}$ and $\varphi_{s0}$ represent the initial phase of the reference signal.

The demodulation algorithm decomposes the modulated signal into four reference signals in Equation (6) by iterative calculation, and calculates the amplitudes of the reference signals. The iteration result is expressed by the following four variables:

(7) $$w\left(k\right)=\left(w_{1}\left(k\right),\;w_{2}\left(k\right),w_{3}\left(k\right),w_{4}\left(k\right)\right).$$

And the iteration process is performed according to the following formulas:

(8) $$r\left(k\right)=\left(r_{1},r_{2},r_{3},r_{4}\right),\;y\left(k\right)=w\left(k\right)\cdot r{\left(k\right)}^{T},\;err\left(k\right)=s\left(k\right)-y\left(k\right),\;w\left(k+1\right)=w\left(k\right)+2\mu \cdot err\left(k\right)\cdot r\left(k\right).$$

where μ is the step factor used for parameter updating, k is the number of sampling points, s(k) indicates the sampling signal, y(k) is the calculated reference signal and err(k) represents the error between the sampling signal and the reference signal.

According to the iterative calculation result of w(k), the signal amplitude A in Equation (1) can be obtained through following equation:

(9) $$A^{2}\left(k\right)=w_{1}^{2}\left(k\right)+w_{2}^{2}\left(k\right)+w_{3}^{2}\left(k\right)+w_{4}^{2}\left(k\right).$$

The data path described above is based on a twice modulated sampling signal, and the reference signal is also modulated. When the sampling signal is modulated only once and has the form of Equation (2), the circuit parameters can be configured through software. When the values of $\omega_{s}$ and $\varphi_{s0}$ are set to 0, the reference signal only contains the unmodulated sine and cosine signals. The path has also been simplified, and the simulation of the simplified data path has been carried out in previous work [16]. In this way, the demodulation of modulated signals of any order can be achieved on the circuit level.

**Figure 6:**
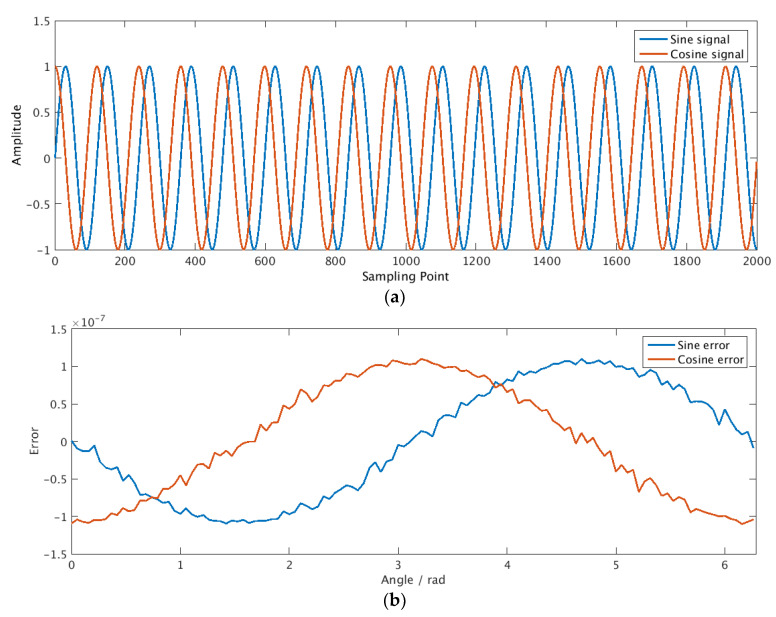
Digital reference signals. (**a**) Waveforms of sine and cosine signals; (**b**) error of digital reference signal.

The mathematical calculations involved in the above scheme are implemented by using combinatorial logic to realize an iterative calculation in a single clock cycle. Based on Cordic algorithm [28], the signal generator is implemented in combination logic. After 28 iterations, it can complete the calculation and output the sine and cosine signal in one clock cycle. The output result is shown in Figure 6, and the error is less than $1.5\times 10^{-7}$.

**Figure 7:**
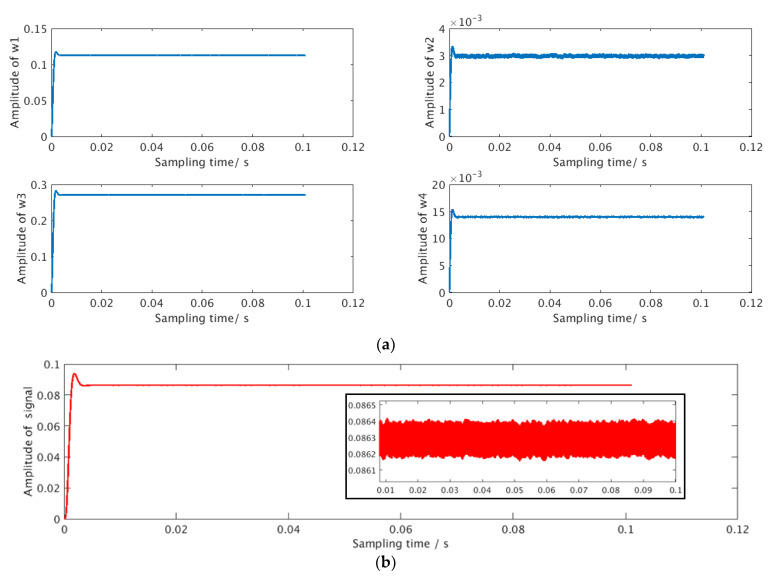
Simulation results for digital demodulation. (**a**) Convergence process of reference signal amplitude components; (**b**) the simulated sensor signal.

Digital simulation was performed on the data from the analog readout circuit. The simulation tool is VCS from Synopsys and the simulated input data is shown in Figure 4 in Section 3. In the simulation, $\omega_{c}=10,000,$ $\omega_{s}=100,000,$ $\varphi_{c0}=-0.2171,$ $\varphi_{s0}=0.08,$ μ=0.03, and related parameters were quantified into fixed-point numbers and configured into the circuit through software. The demodulation results are shown in Figure 7. Figure 7a shows the convergence process of the amplitude components for the four reference signals, and Figure 7b shows the calculation result of the sensor measurement signal.

The circuit parameters of the demodulation module, such as the configuration of frequency, phase, step factor, etc., are completed by a program running in the processor, and the demodulation result can also be sent out of the chip through the processor and the communication interface. The digital part also includes the generation of drive signals, which is realized by a signal generator to generate a sine and cosine signal, and the configuration of the frequency, phase and amplitude of the drive signal is realized through software.

When the measured sensor signal is modulated only once, as shown in Equation (2), the signal generator in the circuit can be set by software to generate a reference signal with a frequency of 0 Hz, which can achieve the compatibility of the demodulation algorithm. The related simulation experiments have been reported in the previous works [15,16].

## 5. Performance and Function Testing of the Chip

### 5.1. Test Results of the Chip

**Figure 8:**
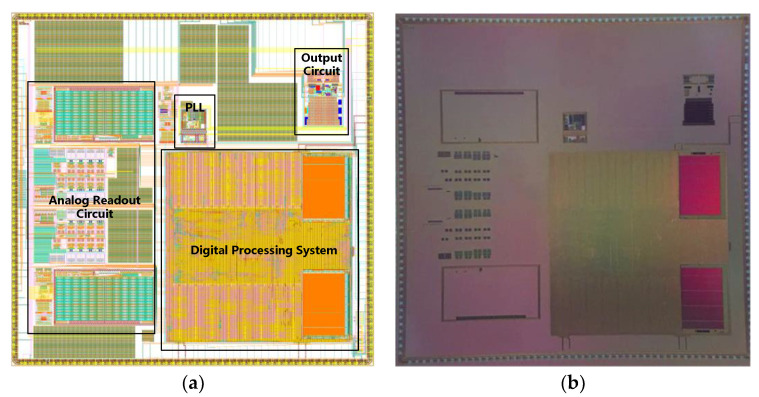
The layout and the die piecture of the designed SoC. (**a**) Chip layout; (**b**) die picture.

The designed SoC was taped out under the SMIC 180 nm CMOS technology. The layout and the die picture of the chip are shown in Figure 8, in which the main parts are marked out. The total area of the chip is $8.9\times 8.7=77.43{\;\mathrm{mm}}^{2}$. The maximum running frequency of the chip is 105 MHz. The analog power supply and digital power supply of the chip are separated. A 5 V voltage source is provided for the analog circuit, and the digital circuit needs a 1.8 V voltage source. When the clock frequency is 51.2 MHz, a processing algorithm was run in the processor to evaluate the power consumption. The currents of the analog and digital circuits are 80 mA and 30 mA, and the power consumption is 400 mW and 54 mW respectively. When the chip is in the power-on idle state, the static current of the digital circuit is 10 mA, and the static power consumption is 18 mW.

From the power consumption results, the digital system has the characteristics of low power consumption, while the power consumption of the analog circuit is large compared with state-of-the-art works. There is a certain difference between the power consumption in analog circuit tested in experiment and the results from simulation and previous works in the laboratory. After analysis and circuit inspection, such test results are related to the on-chip reference voltage generation circuits used for ADC and DAC, and the excessive operational amplifiers. The circuit parameters without proper optimization also have an impact on the power consumption in the analog circuit.

**Figure 9:**
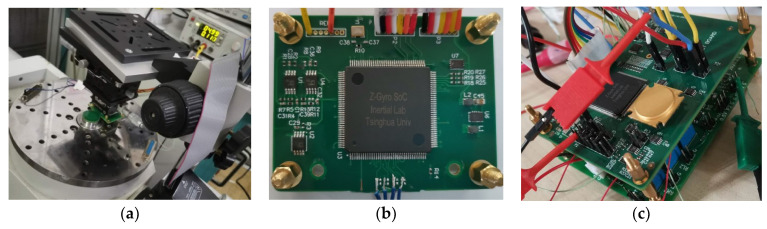
The testing site and testing boards. (**a**) Equipments for function testing; (**b**) step test of an angular displacement sensor; (**c**) frequency sweep experiment of a MEMS gyroscope.

In order to verify the chip’s ability to process signals in the form of Equations (1) and (2), the frequency sweep test of a gyro and the step test of an angular displacement sensor were carried out. The experimental site and the circuit boards involved are shown in Figure 9. The demodulation of the angular displacement sensor needs to obtain the sine signal and the cosine signal from the modulated sensor signals and calculate the angle value. The frequency sweep experiment of the gyro requires the chip to provide excitation signals of different frequencies for the drive axis and the detection axis of the gyro, and demodulate the signal amplitudes to find the resonance frequency corresponding to the maximum amplitude according to the frequency sweep curve.

**Figure 10:**
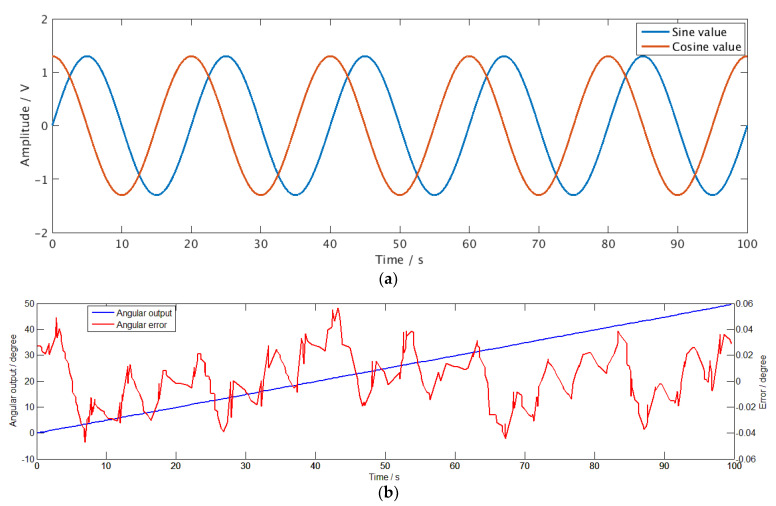
The testing site and testing boards. (**a**) Equipments for function testing; (**b**) step test of an angular displacement sensor.

The output signal of the angular displacement sensor [3] used in the test has a periodicity with a period of 10°. During the experiment, the turntable rotates at a speed of 0.5° per second, and the measurement time is 100 s. The demodulation and angle calculation results from the chip are shown in Figure 10. Since the angle to be measured changes with time, the two demodulated signals are a sine signal and a cosine signal, as shown in Figure 10a. The angular output and error results are plotted in Figure 10b using dual coordinate axes.

**Figure 11:**
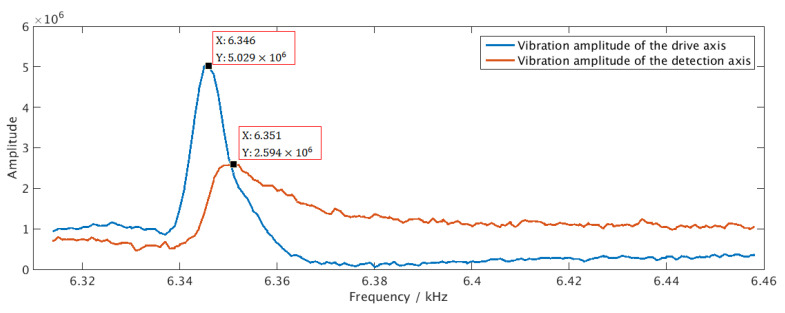
Sweep frequency curves of the drive axis and detection axis of the gyro.

The chip was also electrically connected to an MEMS gyroscope [29] on the PCB, as shown in Figure 9c. The resonant frequency of the gyroscope drive axis and the detection axis is measured in the open-loop, non-tuning state. The digital circuit is configured through an algorithm running in the processor to generate drive signals and reference signals of different frequencies, and the demodulation results are sent through UART. Different demodulation results were collected by adjusting the frequency value. The maximum value of the demodulation result corresponds to the resonance frequency of the gyro. The frequency sweep experiment was performed on both the drive axis and the detection aixs, and the frequency sweep curve is shown in Figure 11. The ordinate represents the numerical value of the demodulation result, which is multiplied by the scaling factor to obtain the true output of the gyro. The scale factor is related to the sensor and needs to be obtained by further sensor experiments. From Figure 11, the experimental results show that the resonant frequency of the measured gyroscope’s drive axis and the detection axis differ by about 5 Hz, which is basically consistent with the measurement results of the same batch of gyroscopes.

The above two tests verified the correctness of the main measurement and control functions of the chip. Other functions of the chip have also been tested. On-chip interface circuit used to drive other AD/DA chips, UART interface circuit, SPI interface circuit, program loading test from off-chip FLASH or on-chip OTP memory, JTAG debugging interface circuit, on-chip single-cycle trigonometric function calculation circuit and other functions also have been verified.

### 5.2. Discussion

The realization of integrated circuits for processing circuits is a means and development trend to further realize high integration and low power consumption in the field of inertial measurement. The measurement and control chip reported in this article is the first monolithic integrated, sensor-compatible measurement and control system that has appeared in recent years. The test results show that the designed and implemented digital-analog hybrid SoC can work normally. Different sensor experiments have further verified the compatibility of the chip in control and processing when the signal of the capacitive sensor meets a specific form. In terms of power consumption, the power consumption of digital systems is relatively low, while the design of analog circuits needs to be further optimized to meet low-power usage scenarios.

The reference signals required by the ADC and DAC in the analog circuit are generated by the internal reference power circuit. This part of the power consumption is relatively large, and this part will be removed in the future design. In addition, there are many operational amplifiers in the circuit, and the static power consumption is relatively large. The use of operational amplifiers will also be optimized. In this design, the interface circuit requires many voltage sources. In the future design, passive circuit implementation schemes will be combined to minimize the use of voltage sources. According to the previously reported power consumption of a single analog interface chip [6,8], a power consumption of tens of mW of analog circuits, and a total power consumption of no more than 100 mW, is the next expected goal. During the experiment, the analog circuit needs external reference capacitors and resistors. When the values of the electronic devices don’t match the internal values, it will cause the mismatch of the circuit parameters, so that the signal input from the ADC to the digital system contains interference signals and no longer meets Equation (1). When the sensor has a large processing error, the preset value of the cancellation capacitance does not match the actual value, and the above problem will also occur. This situation is common in practical applications and will be solved by digital signal processing algorithms in future optimization designs.

## 6. Conclusions

This article reports the design and test results of a digital-analog hybrid measurement and control chip used for capacitive sensors in the field of inertial measurement. The reported chip monolithically integrates the analog interface circuit and the digital processing system, and has the characteristics of miniaturization and low power consumption of the digital system. Benefiting from an on-chip processor and the digital demodulation algorithm compatible with the signal model, the chip has high flexibility in use and can meet different usage scenarios and sensor measurement.

In the integrated measurement and control technology of inertial sensors, the chip reported in this article has a significant improvement in circuit integration and richness of functions compared to previous work [22]. Future research will focus on the power consumption optimization of analog circuits, system-in-package of MEMS structures and SoC chips. Combining high-performance processors and digital signal processing algorithms to further improve sensor accuracy is another research interest.

## Data Availability

Data sharing not applicable.
